# Applying Emmert’s Law to the Poggendorff illusion

**DOI:** 10.3389/fnhum.2015.00531

**Published:** 2015-10-16

**Authors:** Umur Talasli, Asli Bahar Inan

**Affiliations:** Department of Psychology, Atilim UniversityAnkara, Turkey

**Keywords:** Poggendorff illusion, Emmert’s Law, geometric illusions, occlusion illusion, collinearity

## Abstract

The Poggendorff illusion was approached with a novel perspective, that of applying Emmert’s Law to the situation. The extensities between the verticals and the transversals happen to be absolutely equal in retinal image size, whereas the registered distance for the verticals must be smaller than that of the transversals due to the fact that the former is assumed to occlude the latter. This combination of facts calls for the operation of Emmert’s Law, which results in the shrinkage of the occluding space between the verticals. Since the retinal image shows the transversals to be in contact with the verticals, the shrinkage must drag the transversals inwards in the cortical representation in order to eliminate the gaps. Such dragging of the transversals produces the illusory misalignment, which is a dictation of geometry. Some of the consequences of this new explanation were tested in four different experiments. In Experiment 1, a new illusion, the tilting of an occluded continuation of an oblique line, was predicted and achieved. In Experiments 2 and 3, perceived nearness of the occluding entity was manipulated via texture density variations and the predicted misalignment variations were confirmed by using a between-subjects and within-subjects designs, respectively. In Experiment 4, tilting of the occluded segment of the transversal was found to vary in the predicted direction as a result of being accompanied by the same texture cues used in Experiments 2 and 3.

## Introduction

**Figure 1:**
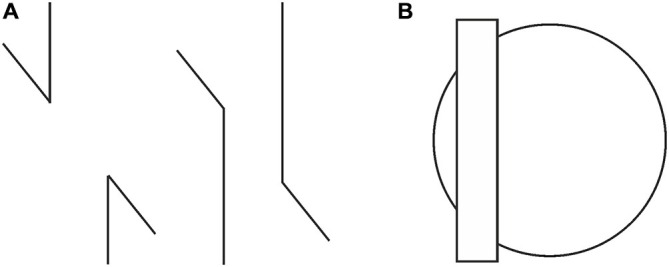
**(A)** The Poggendorff illusion is absent with acute angles (left), but present with obtuse ones (right). **(B)** The presence of the Poggendorff illusion despite the absence of receding planes.

The Poggendorff illusion, which can be described as perceived non-collinearity of the two segments of a physically collinear but interrupted oblique line, continues to defy full explanation for more than a century (Day, 2010). Numerous accounts were offered but each one seemed to fare well with some instances of the basic illusory configuration, failing badly with some other variants. For example, the theory based on misperception of angles in the stimulus (Blakemore et al., 1970; Hotopf and Ollerearnshaw, 1972; Hotopf and Hibberd, 1989) cannot explain why the illusion is abolished when only the acute components are shown as in Figure 1A (Weintraub and Krantz, 1971; Pressey and Sweeney, 1972; Weintraub and Tong, 1974; Day and Dickinson, 1976).

**Figure 2:**
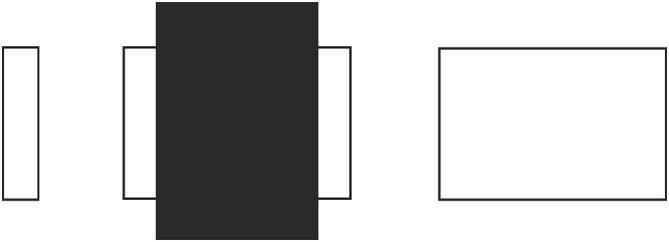
**Paradoxical shrinkage and expansion as a result of occlusion.** The occluded rectangle appears smaller than its unoccluded counterpart on the right, but the visible portion of the occluded rectangle appears larger than its unoccluded counterpart on the left.

Similarly, an entirely different approach, “depth-processing theory” (Gillam, 1971; Spehar and Gillam, 2002; Koning and van Lier, 2007), which involves the impression of receding planes and attributes the illusion to the placement of the upper transversal higher up in the field, fails to account for the illusion occurring with circles as shown in Figure 1B (Schiffman, 2000). Although the circular configuration does not contain any cues serving the depth impression necessary for that theory, the illusion still persists. Yet another significant attempt, Zanuttini’s (1976) notion of “shrinking amodal space” (the distance between the transversals), runs into problems with findings in other areas of the literature (Trueman and Wilson, 1989). More specifically, this approach was inspired by Kanizsa’s (1975) observation that occluded entities appear to shrink in size, but as Palmer et al. (2007) and Vezzani (1999) noted, such objects do just the opposite, that is, expand in size, as seen in Figure 2. Indeed, the visible parts of the occluded entity look much larger than their equivalents placed next to them. Such a contradictory perceptual outcome (i.e., the occluded entity shrinks as a whole while its visible parts appear to expand) poses grave problems in serving as a starting point for explaining the Poggendorff effect via shrinkage of amodal space. Yet another approach involving the notion of shrinkage is the shrinkage of the “modal space” (the distance between the vertical lines; Tong and Weintraub, 1974; Wilson and Pressey, 1976; Day, 1977; Greist-Bousquet and Schiffman, 1985, 1986, 1987; Greist-Bousquet et al., 1989; Weintraub, 1993). This approach is the most similar to our explanation, which we are going to propose below. However, because these studies do not reveal the causal mechanism of such shrinkage, they end up with inconsistent outcomes such as the shrinkage (Tong and Weintraub, 1974; Wilson and Pressey, 1976; Greist-Bousquet and Schiffman, 1987) and the expansion (Wilson and Pressey, 1976; Greist-Bousquet and Schiffman, 1987) of the same entity. In order to alleviate such complications, this shrinkage should emanate from a reliable, empirically demonstrable, and widely accepted fundamental mechanism of perception. The fact that the Poggendorff stimulus has been modified to produce a huge number of variants with the same illusory outcome requires an all-encompassing single mechanism for the sake of parsimony. In support of our argument, other researchers too have suggested that the same process may underlie all variants (MacKay and Newbigging, 1977; Day, 2010). However, that single mechanism has not been proposed yet (Day, 2010).

Within the conceptual frame given above, the mechanism of size perception seems extremely relevant to the Poggendorff situation. Starting from quite early times (e.g., Koffka, 1935; Kilpatrick and Ittelson, 1953), the major explanation of size perception has been given through the “size-distance invariance hypothesis” (see Gogel, 1997). According to this, perceived size is determined by a joint contribution of retinal size and perceived distance. The retinal size of an approaching object, for example, expands, and if such expansion is accompanied by an appropriate decrease in perceived distance, then perceived size remains constant. A special case of this general formula, referred to as Emmert’s Law (Emmert, 1881), dictates that for an unchanging retinal size, perceived size is fully determined by perceived distance. It is thereby understood that two objects projecting equal retinal sizes will have different perceived sizes if their perceived distances are different, the nearer one appearing smaller, as verified by Holway and Boring (1941). A widely accepted classical explanation of the Ponzo illusion and the moon-illusion also rests upon this principle (Rock, 1975, 1995). Of special interest for our purposes is the moon illusion where the retinal image size of the zenith moon is not available while one looks at the horizon moon. The retinal image size of the zenith moon is retrieved from memory and compared against the retinal image size of the horizon moon, and upon finding equivalence between the two, the system operates Emmert’s Law, thereby increasing the perceived size of the horizon moon. Such explanation given by Rock (1975, p. 39–47) shows that the two retinal image sizes involved in the perceptual situation need not be present in the retinal information. While, in the Ponzo illusion the two extensities are concurrently available in the retinal information, in the moon illusion one extensity is available, but the other is retrieved from memory. Hence, the moon illusion teaches us that the retinal sizes for the two extensities need not be concurrently present for Emmert’s Law to operate. Likewise, our analysis of the Poggendorff situation given below entails two extensities of which one is present in the retinal information, but the other is not due to occlusion. We are giving this special circumstance of the Emmert’s Law prior to our explanation given below, so that the retinal extensity concealed by the occlusion will not lead to a difficulty in the comprehension of our theory.

**Figure 3:**
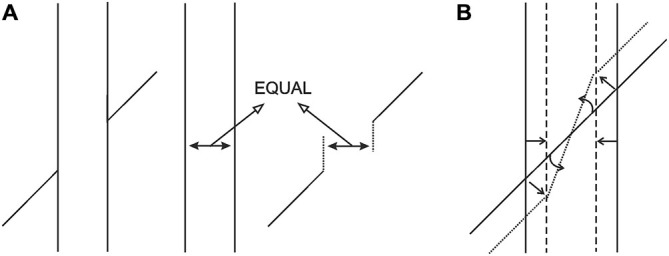
**(A)** The occluding and the occluded extensities are equal in size in the retinal image, but must be registered at different distances due to occlusion. **(B)** Due to Emmert’s Law, the vertical entity, being registered as nearer, shrinks and the occluded oblique extensity is tilted in order to preserve its length and fit into the shrunken space. Finally, the transversals must be dragged obliquely inwards to maintain their contact with the endpoints of the tilted oblique extensity. In this way the gaps produced due to shrinkage in the cortical representation are eliminated, so that the cortical representation will now conform to the absence of gaps in the retinal image.

We propose that the above analysis has a direct bearing on the Poggendorff situation. To make our analysis easier to follow, we will refer to what has been called as the “amodal space” and the “modal space” in the literature up to now as the “occluded space” and the “occluding space,” respectively. Our proposal can be itemized as follows: (1) As can be seen in Figure 3A, the typical Poggendorff stimulus entails two extensities that are identical in retinal size. One of these extensities is the “occluded space,” which is the extent of the separation between the two oblique line segments (i.e., the transversals), and the other extensity, the “occluding space,” is the horizontal extent between the two vertical lines in the classical version; (2) In the typical Poggendorff stimulus, the two oblique lines are accepted as the visible segments of a single line presumably covered or occluded by an entity defined by the vertical lines (Schiffman, 2000; Matlin, 2009). Therefore, the extensity between the verticals “occludes” the transversals; (3) With no exception, the physical reality about the occluding component is that it is always nearer than the occluded one, and therefore must be registered as so; (4) Application of Emmert’s Law to this situation, i.e., unequal perceived distances accompanying equal retinal sizes, dictates a shrinkage of the extensity between the verticals because that component is the occluding one and is therefore nearer; (5) As can be seen in Figure 3B, the shrinkage in question poses a problem for the perceptual system: the oblique extensity between the transversals, which is assumed to be occluded, has a certain length in the retinal image that must be preserved in the cortical representation. Naturally, the system’s solution for implementing shrinkage together with preservation of the length of the oblique extensity requires tilting of this extensity (a notion that is clearly supported in Experiment 1); (6) Another feature of the retinal information that must be preserved is the fact that the transversals are in contact with the verticals. The shrinkage in the cortical representation ought to produce gaps that are unacceptable in view of the retinal information. Hence, as can be seen in Figure 3B, such shrinkage must “drag” the transversals obliquely inwards because this is the only way to maintain the contact with the tilted occluded extensity; and (7) Dragging of the transversals inwards causes the upward and downward displacements of the right and left transversals, respectively, just as would happen in an actual drawing (an obvious dictation of geometry). This, then, describes how collinearity is lost, which is the essence of the present new explanation.

Morgan’s (1999) approach to Poggendorff stimulus is similar to ours since he also suggests that the Poggendorff illusion arises from the orientation change of the unseen oblique segment as mentioned in item (5) above. While the mechanism proposed to be responsible for responding to the orientation of the unseen oblique segment in Morgan’s (1999) work are second-stage filters that encode the centroids of spatial features, we propose the operation of Emmert’s Law.

The parsimony provided by our new explanation is clear. Structural properties of the stimulus need not force modifications in our explanation; whether the occluding component is a rectangle or a circle or even the map of a country (Rock, 1975), the same predictions prevail. Further, supporting and counter evidence for previous explanations can be reinterpreted in the light of this new perspective, thereby eliminating the apparent conflicts, contradictions, and various other inconsistencies in the literature.

We tested our explanation in four different experiments. In Experiment 1, a new illusion producing tilting of the occluded segment of a straight line was introduced as a consequence of our proposed explanation and the presence of an illusory tilt was empirically confirmed. In Experiment 2 (by using a between-subjects design), and Experiment 3 (by using a within-subjects design) perceived distance of the occluding entity was manipulated via a pictorial cue that filled in the space between the verticals by varying the density of the texture and the consequent Poggendorff misalignment magnitude was measured. Our expectation was that the nearer the perception of the occluding entity, the greater the amount of misalignment. Such a result should emanate from our shrinkage hypothesis in that the occluding entity perceived as nearer would have to shrink more due to Emmert’s Law and produce greater misalignment as explained before. This expectation was empirically confirmed as well. In Experiment 4, we sought further confirmation for our approach through finding covariance between the magnitude of misalignment and the amount of tilt built into the same stimulus situation. In other words, the extent of the Poggendorff illusion had already been measured in Experiments 2 and 3 with near-appearing and far-appearing occluding entities, which were brick walls. Following these measurements, in Experiment 4, we embedded translucent glass-like panels, which occluded a blurred segment of the transversal similar to the stimulus of Experiment 1. In this way, we equated the nearness and farness of the stimuli in the Poggendorff and tilt measurements because the two effects were accompanied by the same texture densities. Hence the amount of nearness and farness that affects the Poggendorff misalignment was now used to determine the extent of tilt. A possible covariance between the two measures would thus present clear support for our theory, which was also obtained.

## Experiment 1

**Figure 4:**
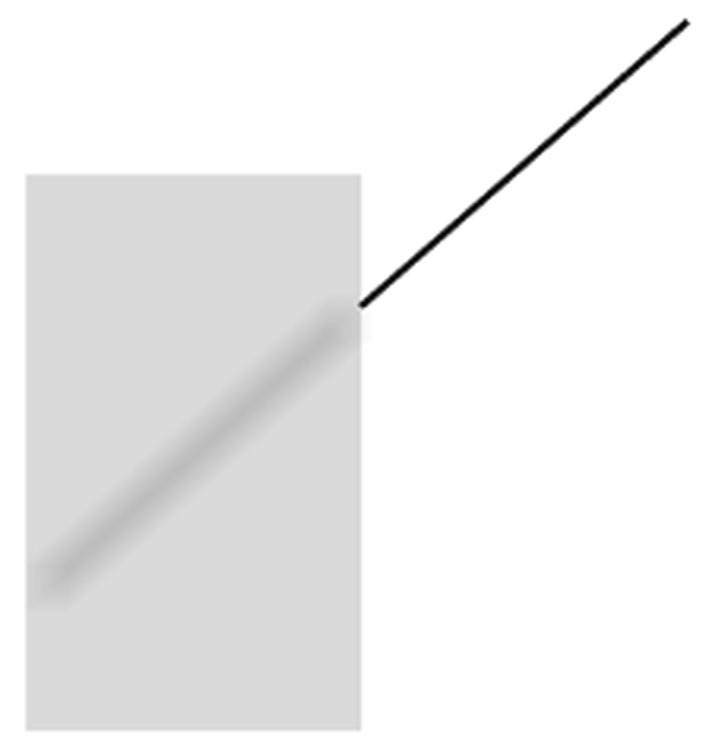
**The stimulus used in Experiment 1.** The transversal continues its path within the verticals, so that the two segments are parts of a straight line. However, the gray area together with the blurred line segment within it creates a strong impression of occlusion, implying a translucent entity in the front. This stimulus creates an illusion of tilt in the blurred segment. In the experiment, participants corrected the apparent tilt to achieve perceived straightness as explained in the text.

**Figure 5:**
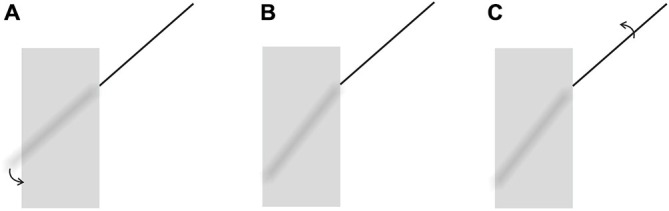
**(A)** Cortical Representation due to the shrinkage of the occluding translucent screen and the necessity of the tilting downwards of the blurred segment. **(B)** The cortical representation after the blurred segment is tilted downwards in order to fit inside the shrunken screen to preserve its length to be in accordance with the retinal image. **(C)** The adjustment the participants make for the collinearity judgment as a result of their illusory perception of the tilt.

Various methods can be devised to test the validity of our new explanation. As a starting point, however, the power of the explanation can be shown by allowing the proposed mechanism to create a “brand new” illusion. Our explanation dictates that the following situation should emanate from the application of Emmert’s Law: as can be seen in Figure 4, the classical Poggendorff stimulus is modified by eliminating the lower transversal and by showing the continuation of the higher transversal in the occluded space. However, the segment traversing the occluded space is blurred in order to give the impression that it is being occluded by a translucent screen. This screen, being the occluding entity, is registered as nearer, and therefore should shrink due to Emmert’s Law (see Figure 3B). Shrinkage of the screen forces two outcomes for the blurred segment: either some fraction of this line should become visible at the two ends without the blur or the absolute length of the blurred line should shorten to fit inside the shrunken screen. Neither of these outcomes is supportable by the retinal image information, and therefore, there remains only one plausible solution, which is preserving its retinal length and tilting itself downwards in order to fit inside the shrunken screen. In line with this logic of perception, our explanation leads to a clear prediction, which is the tilting of the occluded and therefore blurred line segment downwards, thereby introducing a novel illusion, which we will refer to as the “tilt-illusion”. These expected effects are itemized and illustrated in Figure 5. In this experiment, we wanted to see if our explanation is robust enough to be supported by a new illusion, which yields empirically significant results. In addition to this, the obtained tilt illusion also lends support to our theoretical argument that the occluded invisible extensity between the transversals in the Poggendorff stimulus must be tilted as a result of shrinkage (see Figure 3B).

### Method

#### Participants

The sample consists of 68 Atilim University undergraduate students (34 female, 34 male, mean age = 21.7), who volunteered to participate in the experiment.

#### Material

The stimulus used in this experiment is given in Figure 4 and it was drawn using Coreldraw, as all the remaining figures to be presented below in this article. As was mentioned above, the blurred line segment was a straight continuation of the unoccluded segment, which seemed to be occluded by a translucent screen. The dimensions of the figure were as follows: the verticals were 55 mm, the width was 35 mm, the unoccluded and the occluded segments of the transversal were 45 mm each and the thickness of the blurred segment was 3 mm. The angle between the unoccluded segment and the vertical was 50°. This segment made contact with the vertical 12 mm below the upper end, and the gray value for the translucent screen was coreldrw.cpl 20%.

#### Procedure

Atilim University Directive on Human Research Ethics Board approved our study and prior to all the experiments, informed consent of each participant was obtained. Throughout all the experiments, the participants were seated approximately 55 cm from the screen of a 15 inch laptop and they were instructed not to move their body or their head until they completed their settings.

We wanted to measure the magnitude of the tilt illusion in two ways: first, we asked the participants to correct the seemingly tilted, but physically straight line by rotating up the unoccluded segment until it appeared to be a straight continuation of the occluded segment, thus making the necessary correction required by their illusory perception of the tilt. Second, we presented the unoccluded segment as obviously and physically oriented upwards by an amount of nine degrees compared to the occluded segment, and asked the participants to rotate down the unoccluded segment until it appeared as a straight continuation of the occluded one. The point at which the participants stopped the downwards rotation would give us the magnitude of the tilt illusion and would allow us to compare the magnitudes obtained in two different ways. If the magnitudes would turn out to be comparable despite different methods, then we would be more confident about the robustness and the reliability of the tilt illusion.

One half of the participants were thus assigned randomly to the first condition, where they saw the configuration in Figure 4 and were asked if the unoccluded segment and its continuation within the rectangle looked absolutely straight or not. All of the participants perceived the tilt, and the unoccluded segment was rotated upwards by 0.5° steps via pressing of the arrow key until the participant decided that straightness was achieved.

In the second condition, the other half of the participants were shown Figure 4 with the unoccluded segment oriented upwards by 9°. All of the participants saw the obvious and physically present lack of straightness in the stimulus, and were asked to rotate the unoccluded segment downwards by 0.5° steps until they decided that straightness perception was achieved. In both conditions, in case a participant thought he/she exceeded the straightness setting, reversing the direction of rotation was allowed.

### Results

In the first condition, the participants rotated the unoccluded segment from the straightness position upwards by an angle of 3.78° on the average. This meant that the participants saw the occluded segment as tilted downwards by 3.78° although it was physically a straight continuation. Hence, the magnitude of the tilt illusion according to this measurement was obtained by this value.

In the second condition, the participants rotated the unoccluded segment downwards by an angle of 5.25° on the average. At this point, the two segments appeared straight. Had they achieved the correct perception of straightness, they would have had to rotate downwards by an amount of 9°. The fact that they stopped at 5.25° meant that they were subject to the tilt illusion by an amount equivalent to the difference between 9° and 5.25°. This difference turned out to be 3.75°, which is strikingly close to the magnitude obtained in the first condition. Hence the tilt illusion appears to be quite a stable and reliable phenomenon.

A one sample *t*-test was conducted to find out if the magnitude of the tilt was significant to indicate the existence of the illusion. The mean values for the magnitude of the angle of tilt for the first condition (*M* = 3.78°, *SD* = 1.62), for the second condition (*M* = 3.75°, *SD* = 1.50) and across the two conditions, (*M* = 3.765°, *SD* = 1.55) indicated that the line was judged to be straight at an angle significantly different from zero, with *t*-values as follows: *t*_(33)_ = 13.57, *p* < 0.001, *d* = 2.33, for the first condition; *t*_(33)_ = 14.59, *p* < 0.001, *d* = 2.50, for the second condition; *t*_(65)_ = 20.01, *p* < 0.001, *d* = 2.43, for the combined data. The illusion was thus verified by significant magnitudes in all conditions.

### Discussion

In this experiment, we created a new illusion that sprang from our novel explanation of the Poggendorff illusion. In this new illusory situation, the appearance of tilt was induced for the blurred part of the straight line because the blur generated the impression of being occluded by a seemingly translucent screen. The occluding screen was then subject to Emmert’s Law and was consequently supposed to shrink. As a result of such shrinkage, “the fitting problem” was expected to be solved by the perceptual system by tilting the blurred line, since it was the only plausible solution to this problem as explained in the Introduction of Experiment 1. This expectation was fully born out with a significant empirical outcome, which not only produced a new illusion, but also lent support for the Emmert’s Law-based new explanation of the Poggendorff illusion. Further, the results of this tilt illusion can be considered as evidence against the “amodal space shrinkage” explanation (Zanuttini, 1976) for the Poggendorff illusion. If it were the occluded space that shrank, there would be no need for tilting (and therefore no tilt illusion), because the blurred segment would not face a “fitting problem” due to being shortened itself. On the contrary to the explanations of Zanuttini (1976), the occurrence of tilting suggested in a very rational way that what shrank was not the occluded space, but it was the occluding one. One additional benefit of this result is that, the finding of tilting is in support of our proposed mechanism in that the invisible occluded oblique extensity in the Poggendorff stimulus also needs to be tilted to fit into the shrunken space, and thereby, it dictates an “oblique” dragging of the visible transversals inwards to maintain contact with the endpoints (see Figure 3B).

## Experiment 2

In this experiment, we attempted to manipulate the perceived nearness of the occluding vertical entity by providing pictorial information about a familiar surface. We preferred to use the picture of a brick-wall as the familiar surface because it allows variation of perceived distance as a function of variation of retinal image size of the bricks (i.e., texture density). This is so because a typical brick has a stable and well-known physical size, and therefore, any change in its retinal image size will automatically translate to a change of perceived distance. Hence large and small bricks in the picture will get compared against the prototypical brick size in memory and will dictate near and far distances, respectively. The idea here is that when we allow an occluding entity to appear nearer in the Poggendorff stimulus, there will be more shrinkage of the occluding entity, more dragging inwards of the transversals, and consequently, more misalignment. Hence, our specific prediction is that more misalignment should be obtained in the large-brick wall stimulus as compared to the small-brick wall. It should be noted again that we kept the Poggendorff stimulus identical in the two cases (width, length, transversal size and angles, etc.) except that we filled in between the verticals with different texture densities so that the apparent nearness was manipulated. If our proposed mechanism is indeed correct, it should respond to such distance manipulation despite the sameness of other critical Poggendorff stimulus characteristics. In Masini et al.’s (1994) study the space between the verticals was also filled with different textures such as random dot textures of different density. The difference between their study and ours is that their stimulus texture being random dots does not control perceived distance but our stimulus textures being variations of a standard size achieve that control.

### Method

#### Participants

Forty-six university students (23 female and 23 male, mean age = 22.8) from different departments of Atilim University participated in this experiment voluntarily.

#### Material

**Figure 6:**
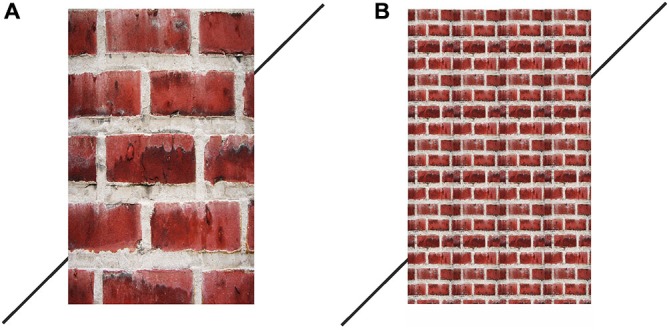
**Brick wall pictures used in the manipulation of perceived nearness of the vertical entity in Experiment 2, as they appear in the aligned position.** As can be observed, the misalignment in the large brick-wall **(A)** appears greater than that in the small brick-wall **(B)**. The panel on the right has been reduced to one fourth of the size of the units in the panel on the left.

The stimuli used in this experiment are given in Figure 6. The dimensions of both brick-wall figures were as follows: (a) for the vertical entities the height was 110 mm and the width was 70 mm; (b) the lower and higher transversals were 37 mm each; (c) the left transversal made contact with the brick wall at 20 mm above the floor-line; (d) the right transversal at the starting point made contact with the brick wall at 15 mm above the floor-line; (e) the angle between the transversals and the verticals were 45-degrees; (f) the average size of a brick in the large-brick wall stimulus was 33 mm in length and 19 mm in height; and (g) the small-brick wall stimulus yielded a greater density by reducing the mentioned unit size to one fourth of those in the large-brick wall stimulus.

#### Procedure

**Figure 7:**
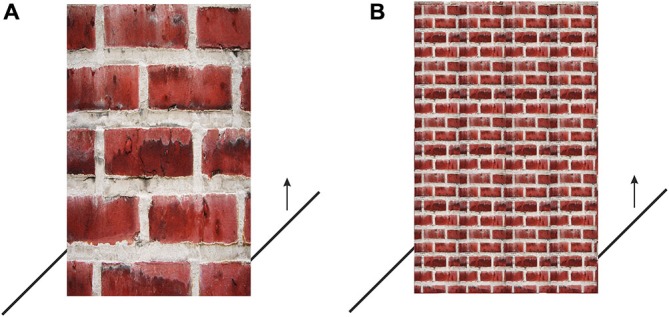
**Large-brick wall stimulus (A) and small-brick wall stimulus (B) presented at the beginning of Experiments 2 and 3 with the right transversal located near the floor-line of the wall.** The participants were instructed to press the up-arrow key on the keyboard until they decided that the right transversal was collinear with the left one.

Half of the participants were presented the large-brick wall stimulus, while the other half were presented the small-brick wall. The figures were presented with the right transversal located near the floor-line of the wall and the participants’ were instructed to press the up-arrow key on the keyboard (see Figure 7) until they decided that the right transversal was collinear with the left one.

### Results

**Figure 8:**
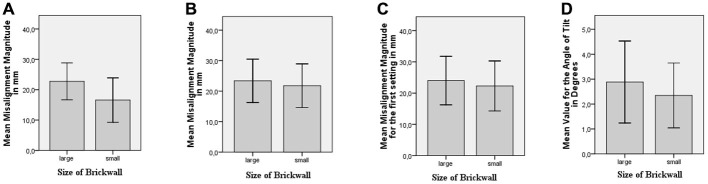
**(A)** Mean value for the magnitude of misalignment for different brick wall sizes for Experiment 2. **(B)** Mean value for the magnitude of misalignment for different brick wall sizes for Experiment 3. **(C)** Mean value for the magnitude of misalignment of the first setting for different brick wall sizes for Experiment 3. **(D)** Mean value for the angle of tilt for different brick wall sizes for Experiment 4.

Six of the participants’ data were removed from the analysis, since they made either settings above 40 mm or invalid settings. There was a difference of 6.1 mm in the predicted direction between the mean value for the magnitude of misalignment for the large-brick wall and the small-brick wall. An independent samples *t*-test was conducted to find out whether this difference was significant. The mean value for the magnitude of misalignment for the large-brick wall (*M* = 22.7 mm, *SD* = 6.1) was significantly greater than the mean value for the magnitude of misalignment for the small-brick wall (*M* = 16.6 mm, *SD* = 7.3); *t*_(38)_ = 2.89, *p* = 0.003, *d* = 0.91 (see Figure 8A).

### Discussion

The results of this second experiment clearly gave further support to the Emmert’s Law-based explanation of the Poggendorff illusion. Specifically, our attempt to manipulate the amount of apparent nearness of the occluding vertical entity via texture density cues of distance found a response in the perceptual system and the nearer the vertical entity appeared, the greater the magnitude of the illusion was. Since the predicted result was obtained, the proposed effect of the apparent distance of the occluding entity on the misalignment magnitude in the Poggendorff illusion was demonstrated.

## Experiment 3

In this experiment, we wanted to confirm that the results we obtained in Experiment 2 are indeed due to the manipulation of the registered distance. Therefore we replicated Experiment 2 by using a within-subjects design, which was followed by participants’ distance estimation for the large and small-brick wall stimuli.

### Method

#### Participants

Thirty-four Department of Psychology students (29 female and 5 male, mean age = 22.4) from Atilim University participated in this experiment in exchange of course credit.

#### Material

**Figure 9:**
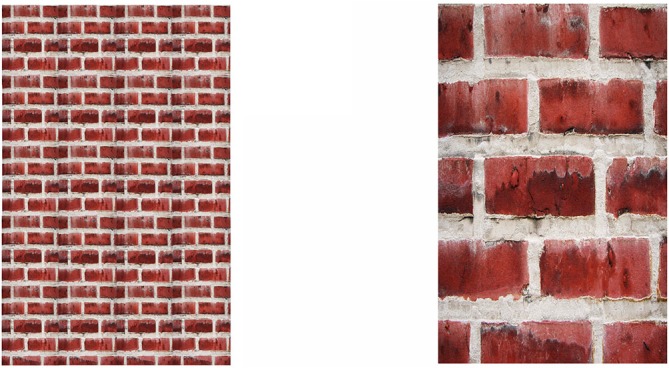
**The brick wall figures presented without the transversals for making distance estimation**.

The stimuli used in this experiment for the misalignment settings are the same as the ones used in Experiment 2 and are given in Figure 7, the stimuli used for the distance estimations are given in Figure 9.

#### Procedure

The participants were presented both the large-brick wall stimulus and the small-brick wall stimulus successively. From each participant four settings were taken. The order of presentation was counterbalanced, such that half of the participants were presented the large-brick wall stimulus first and the small-brick wall stimulus next, followed by the same order of settings once more. While the other half were presented the small-brick wall stimulus first and the large-brick wall stimulus next, followed by the same order of settings once more. The procedure otherwise was the same as the procedure of Experiment 2’s. After the participants completed the four settings, they were presented the brick wall figures without the transversals, as given in Figure 9, and were asked to make distance estimation for these figures. Then finally they were asked which one of the brick walls, large or small, appears farther.

### Results

Among 34 participants, 27 of them said that the small-brick wall was more distant than the large-brick wall, three of them said that they were at the same distance, while four of them said that the large-brick wall was farther. One of the participants, considered as an outlier was removed from the analysis, due to making a setting of above 45 mm.

For each participant, the mean misalignment for the large-brick wall and the small-brick wall was calculated by using the two settings for each. There was a difference of 1.61 mm in the predicted direction between the mean value for the magnitude of misalignment for the large-brick wall and the small-brick wall. A paired samples *t*-test was conducted to find out whether the difference in misalignment magnitude for the small vs. large-brick wall was significant. The mean value for the magnitude of misalignment for the large-brick wall (*M* = 23.4 mm, *SD* = 7.1) was significantly greater than the mean value for the magnitude of misalignment for the small-brick wall (*M* = 21.8 mm, *SD* = 7.2); *t*_(32)_ = 2.55, *p* = 0.008, *d* = 0.44 (see Figure 8B).

When only the first settings were considered, there was a difference of 1.72 mm for the mean value for the magnitude of misalignment for the large-brick wall and the small-brick wall in the predicted direction. The results of the paired samples *t*-test showed that this difference was significant. The mean value for the magnitude of misalignment of the first setting for large-brick wall (*M* = 24.0 mm, *SD* = 7.8) was significantly greater than the mean value for the magnitude of misalignment of the first setting for the small-brick wall (*M* = 22.3 mm, *SD* = 8.0); *t*_(32)_ = 2.11, *p* = 0.022, *d* = 0.37 (see Figure 8C).

### Discussion

Firstly, when participants’ distance estimations for the large and small-brick wall stimuli are considered, the results of Experiment 3 demonstrated that our attempt in manipulating the apparent distance by using the large and small-brick wall stimuli was successful. Secondly, the results we obtained for Experiment 2 by using a between-subjects design was replicated by using a within-subjects design. In other words, the apparent distance of the occluding entity, the brick-walls, affected the Poggendorff misalignment magnitude in the predicted direction.

## Experiment 4

In Experiment 4, we attempted to test the second aspect of our theory. The first aspect is the effect of apparent distance of the occluding entity on the amount of misalignment, which was shown in Experiments 2 and 3. The second aspect involves the prediction that a greater amount of misalignment is accompanied by a greater amount of tilt of the occluded entity. In Experiment 1, we already verified that the occluded oblique segment between the transversals was subject to tilting, and in the present experiment we attempted to show that this tilting increased together with the amount of misalignment. Since we already showed that the Poggendorff illusion responds to apparent nearness in the predicted direction via texture density manipulations, we chose to test the second aspect of our theory regarding different amounts of tilt within the very same stimulus situation. That is, in order to ensure the equivalence of apparent distance effects for the misalignment and the tilt, we embedded a translucent glass panel occluding a blurred segment of the upper transversal within the same large and small brick-walls in order to see if apparent nearness that affects the Poggendorff misalignment also affects the amount of tilt in the predicted direction. It should be noted that a difference in the amount of tilt in two separate apparent distances is not easy to obtain because we are not trying to show the presence of tilt (as in Experiment 1) anymore, but we are after demonstrating a difference within a subtle effect.

### Method

#### Participants

Twenty-six undergraduate students (15 female, 11 male, mean age = 20.9) of Atilim University participated in this experiment voluntarily.

#### Material

**Figure 10:**
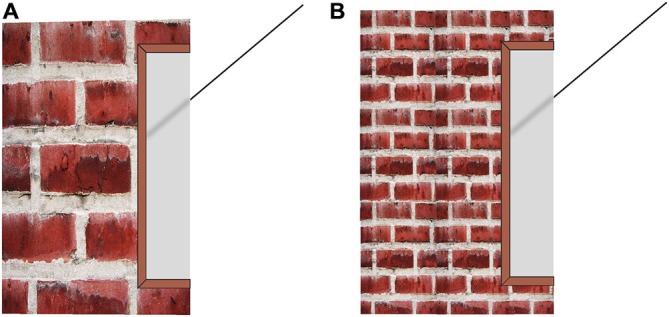
**The large (A) and small (B) brick wall stimuli in Experiment 4 with embedded translucent glass panels occluding the continuation of the upper transversal.** The perceived distance of the glass panel and the brick wall in each version was equated due to embedding.

The stimuli used in this experiment are given in Figure 10. The dimensions and the texture densities of the brick-wall figures were the same as in Experiment 2, except that the lower transversal was absent because it was unnecessary, since we were not measuring the misalignment. The length of the unoccluded segment of the transversal was 55 mm, and the occluded and therefore blurred segment was 25 mm. The translucent glass panel embedded as vertically centered in the brick-wall was 15 mm in width and 83 mm in height. The gray value of the panel and the width of the blurred segment were the same as in Experiment 1. The transversal made contact with the glass panel at 80 mm above the floor-line. When the transversal made a 50-degree angle with the vertical, it became physically a straight continuation of the occluded segment (although it did not appear so, as can be seen in Figure 10).

#### Procedure

The participants were presented each version of Figure 10 in which the starting position of the unoccluded segment made an angle of 40° with the vertical, in other words, 10° upwards from the physical straightness position. All of the participants saw the obvious and physically present lack of straightness in the stimulus and they were asked to press the downward arrow key to rotate the unoccluded segment downwards by 0.5° steps until they decided that straightness was achieved.

Data were collected within-participants with the order of large and small-brick presentations counterbalanced. Since this experiment aimed to find a subtle difference measurement, inter-participant variation in the perceived distance of the large and small brick-walls was thought to be a potential handicap. For the sake of reducing such inter-participant variation, a within participants comparison was thought to be more appropriate.

### Results

The mean value for the angle of tilt in the large-brick wall was *M* = 2.88 °, *SD* = 1.65 and the mean value for the angle of tilt in the small-brick wall was *M* = 2.35 °, *SD* = 1.30 (see Figure 8D). A paired-samples *t*-test was conducted, which indicated that this difference was significant; *t*_(25)_ = 2.29, *p* = 0.015, *d* = 0.45.

### Discussion

The results of Experiment 4 showed that the amount of tilt accompanied by texture density manipulations changed as a function of apparent distance. Specifically, the near-appearing surface yielded a significantly greater amount of tilt as compared to the far-appearing surface. This finding is in harmony with our theory in that apparent nearness of the occluding surface not only increases the magnitude of misalignment (as found in Experiments 2 and 3), but it also increases the perceived amount of tilt of the occluded segment. With respect to these two phenomena, our theory proposes a common underlying mechanism, which is the shrinkage of the occluding surface due to the operation of Emmert’s Law, and the finding that misalignment and tilt vary together as a function of apparent distance lends clear support to both aspects of our theory.

## General Discussion

Our analysis of studies in the literature suggests a number of points that can be made regarding the Poggendorff illusion. First, we would like to note that the Poggendorff mystery is not explainable by a simple neurology-based bottom-up approach. Even at the neurological level of analysis, the complexity of the mechanism is suggested by the evidence for top-down influences (Liao et al., 2011; Medvedev et al., 2011). That the Poggendorff illusion is unaffected by right or left hemisphere lesions (Grabowska et al., 1992) also indicates that neural infrastructure is not sufficient for an explanation. Further, the complexity of the mechanism is shown even at the neural level by the dominance of occluding (modal) completion over the occluded (amodal) one, when both are present in the same stimulus (Brodeur et al., 2009). The findings of Brodeur et al. (2009) suggest that the neural system treats the occluding and the occluded spaces differentially, which is relevant to our argument of the shrinkage of the occluding space. In relevance also is the recent literature involving fMRI studies which are also supportive of our argument in that neural correlates for size constancy and Emmert’s Law have been discovered (e.g., Murray et al., 2006; Sperandio et al., 2012). In this way, recent neural studies have begun to show that a simple bottom-up analysis is not sufficient for an in-depth understanding of visual illusions that involve size-distance scaling.

Of special importance for our explanation are findings regarding stereoscopic manipulations. For example, Takeichi and Nakazawa (1994) demonstrated that as transversals were brought nearer stereoscopically the misalignment sharply decreased. This finding suits our explanation because, the verticals now being behind cannot occlude the transversals and therefore the underlying assumption of occlusion in the Poggendorff illusion is not present. In further support, Wang and Idesawa (2004) showed that the Poggendorff illusion occurred only when the transversals were stereoscopically perceived as farther than or equal to the distance of the verticals (also see Koning and van Lier, 2007). These findings are also in line with our explanation. More specifically, when transversals are stereoscopically equidistant with or pushed away from the verticals, the underlying assumption of occlusion in the Poggendorff illusion is maintained, so the illusion prevails.

**Figure 11:**
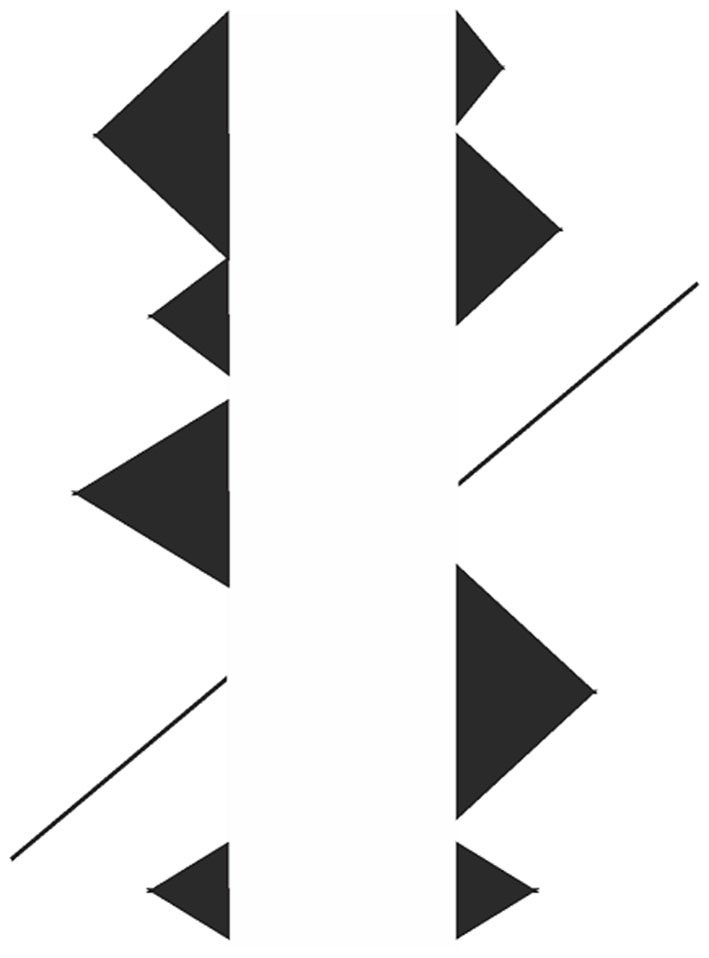
**A full blown Poggendorff illusion occurring in a typical Kanizsa figure where implied occlusion by the white rectangle creates subjective contours.** An example showing that the illusion can occur without actual contours and angles.

After showing that our explanation is in congruence with even the stereoscopic manipulations, which are rather rare and exceptional, we would like to discuss how we meet the challenge of accounting for the classical variants of the Poggendorff illusion, which all depend on 2-D configurations. An especially intriguing one among the classical variants is the acute vs. obtuse-angle versions, with no effect in the former and sizable effect in the latter (Rock, 1995, p. 162). According to our theory, the differential effects found here have nothing to do with angles. As can be seen in Figure 1A, a major difference between the configurations of the two versions is that the obtuse-angle version displays a partial overlap of the vertical segments, whereas the acute-angle version has no overlap. What we mean by overlap is the simultaneous lateral existence of parts of the vertical segments, giving the impression of an occluding vertical entity, especially when one considers the possibility that the visible vertical segments may be continuing as subjective contours. Such “implied occlusion” is manifested in another variant (see Figure 11), which is similar to one given by Rock (1995, p.163), where implied occlusion leading to subjective contours is sufficient to produce a full blown Poggendorff illusion in the absence of verticals and angles. While implied occlusion present in both the obtuse-angle version and the Kanizsa stimulus in Figure 11, calls for the operation of Emmert’s Law and the already explained consequences leading to misalignment, the absence of implied occlusion for the acute-angle version prevents Emmert’s Law from playing a role in the situation and therefore the illusion is completely eliminated.

**Figure 12:**
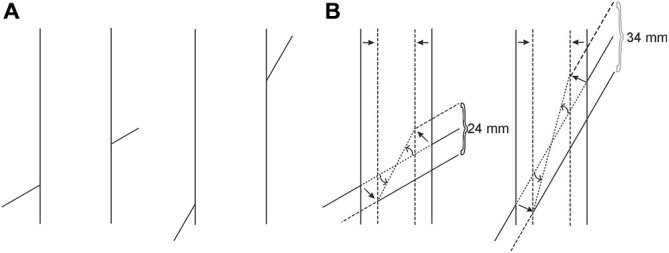
**(A)** One of the classical variants of the Poggendorff illusion, where misalignment is a function of increasing steepness of the transversals. **(B)** The graphic representation based on our theory, demonstrating why this functional relationship should hold.

The increase of misalignment as a function of increasing steepness of the transversals (Figure 12A) is another challenging classical variant, which can be explained by our theory as follows: the important aspect of steepness is that steeper transversals provide longer occluded oblique extensities, which traverse between the transversals’ endpoints. Therefore, as can be seen in Figure 12B, in order to fit a longer occluded extensity into the same shrunken space, the extent of tilt must be greater. This, then, leads to a greater amount of displacement of the transversals up and down as they are dragged obliquely inwards to preserve the contact with the verticals. In this way, and again as a dictation of geometry, misalignment is an increasing function of the transversals’ steepness.

**Figure 13:**
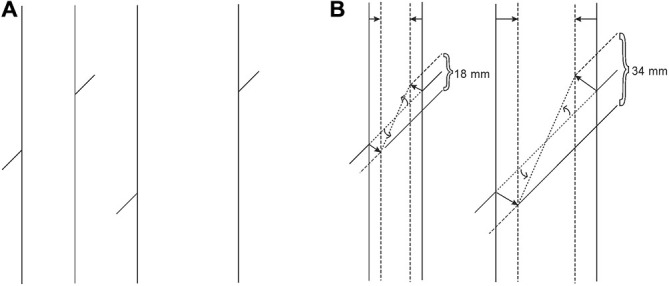
**(A)** One of the classical variants of the Poggendorff illusion, where misalignment is a function of increasing width of the vertical entity. **(B)** The graphic representation based on our theory, demonstrating why this functional relationship should hold. The increase in shrinkage is drawn as proportionate to the increase in width.

Yet another variant, which yields increasing misalignment as a function of increasing width of the occluding entity (see Figure 13A) is explainable by the same geometric dictation plus the following assumption of the perceptual system: especially in impoverished perceptual situations, object size is known to be a cue to distance. A wider occluding entity is often registered as nearer than a narrower one, and therefore the amount of shrinkage is greater for a wider entity, as can be seen in Figure 13B. Consequently, therefore, the geometric dictation comes into play at this point and because the transversals are being dragged to a greater extent, the magnitude of the misalignment is increased.

There are two more puzzling classical variants, which also can be handled by our explanation. As was mentioned in the Introduction section, the first of these is a circle partly occluded by a rectangle (Schiffman, 2000), where, as can be seen in Figure 1B the arc to the left of the rectangle appears to be part of a smaller circle, even though it is a segment of the one and the only circle in the figure. Although this figure does not seem to be a typical Poggendorff variant, the small arc appears smaller as an artifact of the misalignment of the two arcs’ terminal ends. Our explanation again is that, the shrinkage of the occluding rectangle drags the two arcs inwards, so that the resulting misalignment dictates that the two arcs belong to two circles of different sizes.

**Figure 14:**
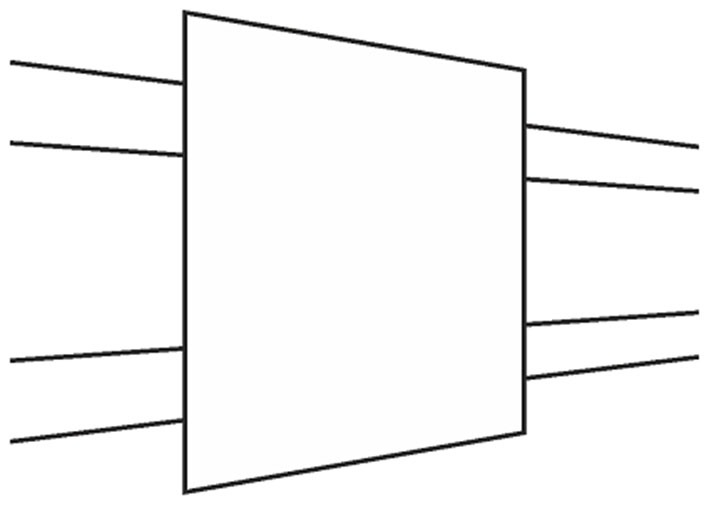
**Perspective injected into the occluding entity attenuates the illusion because drawing the figure in accordance with proper perspective rules severely limits the apparent nearness of the occluding entity (as explained in the text).** So this produces very little shrinkage and results in the attenuation of the illusion.

The variant in Figure 14 involves perspective (Gillam, 1971; Spehar and Gillam, 2002; Koning and van Lier, 2007). It is obvious that perspective injected into the occluding entity greatly attenuates the misalignment. Our explanation is as follows: when the occluding entity lacks perspective information, as in the classical case, there is no limiting factor for the occluding entity to be perceptually brought near. However, when perspective is injected into the occluding entity, the vanishing point that governs the orientations of the transversals also governs the bottom and top edges of the occluding entity, thereby placing this entity at a very small separation in depth from the transversals. This situation places an obvious constraint for the occluding entity to appear perceptually near. Hence, lesser extent of perceptual nearness means lesser extent of shrinkage of the occluding entity, and this means attenuation of the misalignment.

Although the validity of our new explanation is supported by its being congruent with numerous findings in the Poggendorff literature in a parsimonious manner, we sought to find original empirical support, which we believe was achieved in three separate experiments. In Experiment 1, we followed the implications of our theory and produced a new illusion, which we called the “tilt illusion.” In the following two experiments, we preferred to use pictorial cue manipulations well-established in the literature which varied the perceived distance along the *z*-dimension. Accordingly, in Experiment 2, we attempted to manipulate the perceived nearness of the occluding entity via changing the texture density which filled in the space between the two verticals. Thus, the nearer-appearing surface containing larger bricks was expected to be perceived nearer and thereby to increase the amount of misalignment, which was confirmed. In Experiment 3, we further followed the implications of our theory in that the expected increase in the amount of tilt of the occluded oblique segment as a function of perceived nearness was directly tested. We did this by making part of the occluded segment visible through a translucent panel embedded in near and far-appearing brick walls. The expected increase in the amount of tilt in the case of large-brick wall (the near-appearing one) was also obtained.

**Figure 15:**
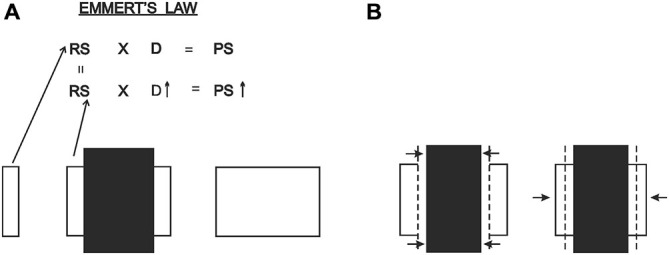
**(A)** Explanation of the paradoxical situation in the occlusion illusion: the expansion of the visible part of the occluded entity vs. its unoccluded counterpart is a dictation of Emmert’s Law, that is, the retinal image sizes are equal, but due to the occlusion the visible part of the large rectangle is registered as being farther, thereby expanding in perceived size. **(B)** On the other hand, the totality of the occluded entity looks smaller than its unoccluded counterpart. According to our theory, this results from the shrinkage of the occluding entity and the dragging in of the two visible parts.

The totality of the work we have presented above points to an important fact: the Poggendorff illusion actually is based upon the so-called occlusion illusion. As was mentioned previously, a fundamental assumption made by the perceptual system for the Poggendorff stimulus is the occlusion of the transversals by the entity indicated by the vertical lines, or any other variation. As was noted by Palmer (1999), a comprehensive explanation for all aspects of the occlusion illusion is still lacking. We touched upon this problem in the Introduction in relation to our criticism of Zanuttini’s (1976) explanation. Actually, as can be seen in Figure 15, our approach can handle the occlusion illusion via Emmert’s Law. First, the apparent enlargement of the visible parts of the occluded entity is due to the greater registered distance of the occluded entity (due to being occluded) as compared to the unoccluded counterpart despite equal retinal image sizes (Figure 15A). As for the perceived shrinkage of the whole occluded entity, our explanation is that the shrinkage of the occluding entity necessitates the dragging inwards of the visible parts in order to eliminate the gaps in the cortical representation for the sake of complying with the retinal information which contains no gaps, as can be seen in Figure 15B. This last treatment shows that our explanation is parsimonious not only within the Poggendorff illusion, but also across two other illusory situations, the occlusion illusion, and our newly presented “tilt” illusion. This is because our explanation is based upon a fundamental perceptual mechanism that underlies a wide range of situations, which trigger nonconscious distance registrations.

In conclusion, we contribute to the understanding of the Poggendorff illusion by applying Emmert’s Law to it, which turned out to show the power of this novel approach by both producing new empirical findings and providing parsimonious explanations for the existing ones, including the elimination of the inconsistencies present in the literature. Our explanatory efforts often reflected a fundamental principle, which we consistently obeyed. This principle is that perceptual outcomes result from the reconciliation of bottom-up and top-down processes. Emmert’s Law entered the situation as a top-down process, which produced a cortical representation that departed from the retinal one. The decision process, in an effort to reconcile the discrepancy between the retinal image and the cortical representation, implemented the so called “dragging” operation, which invited dictations of geometry, resulting in illusory perceptual outcomes. This principle, which is clearly implicated in the Poggendorff situation ought to be more general an account for a rich variety of perceptual situations. We think that this aspect of our study should be quite heuristic and produce a great number of new hypotheses.

## Conflict of Interest Statement

The authors declare that the research was conducted in the absence of any commercial or financial relationships that could be construed as a potential conflict of interest.
